# Crosstalk between heredity and environment in myopia: An overview

**DOI:** 10.1016/j.heliyon.2024.e29715

**Published:** 2024-04-16

**Authors:** Jiawen Hao, Zhaohui Yang, Ruixue Zhang, Zhongyu Ma, Jinpeng Liu, Hongsheng Bi, Dadong Guo

**Affiliations:** a Shandong University of Traditional Chinese Medicine, Jinan, 250002, China; b Affiliated Eye Hospital of Shandong University of Traditional Chinese Medicine, Jinan, 250002, China; c Shandong Provincial Key Laboratory of Integrated Traditional Chinese and Western Medicine for Prevention and Therapy of Ocular Diseases, Jinan, 250002, China; d Shandong Academy of Eye Disease Prevention and Therapy, Jinan, 250002, China; e Shandong Provincial Clinical Research Center of Ophthalmology and Children Visual Impairment Prevention and Control, Jinan, 250002, China; f Shandong Engineering Technology Research Center of Visual Intelligence, Jinan, 250002, China; g Shandong Academy of Health and Myopia Prevention and Control of Children and Adolescents, Jinan, 250002, China; h Medical College of Optometry and Ophthalmology, Shandong University of Traditional Chinese Medicine, Jinan, 250002, China

**Keywords:** Myopia, Heredity, Environment, Interaction

## Abstract

In recent years, the prevalence of myopia has gradually increased, and it has become a significant global public health problem in the 21st century, posing a serious challenge to human eye health. Currently, it is confirmed that the development of myopia is attributed to the combined action of genes and environmental factors. Thus, elucidating the risk factors and pathogenesis of myopia is of great significance for the prevention and control of myopia. To elucidate the impact of gene-environment interaction on myopia, we used the Pubmed database to search for literature related to myopia. Search terms are as follows: myopia, genes, environmental factors, gene-environment interaction, and treatment. This paper reviews the effects of gene and environmental interaction on myopia.

**Key Messages**

1. The development of myopia is affected by genes and environmental factors.
2. Metabolic disorders and hypoxia are involved in myopia.
3. Interaction between genes and the environment can regulate the myopia progression.

## 1 Introduction

Refractive errors are the product of a mismatch between the axial length of the eye and its optical power, creating blurred vision [1]. The most common ametropia is myopia [2]. The definition of myopia is a refractive state in which parallel light passes through the refractive system of the eye and the focus falls in front of the retina. At present, the global prevalence of myopia is about 28.3 %. It is estimated that 50 % of the world's population will be affected by myopia by 2050, and the prevalence of high myopia will reach 10 % [3]. Following the data reported, the prevalence of myopia in young adults in East and Southeast Asia was approximately 80%–90 %, accompanied by a high prevalence of high myopia (10–20 %) [4]. Myopia, especially high myopia (＞-6.00D), is associated with macular degeneration, retinal detachment, and so on [5], which seriously threatens people's life and health. Therefore, it is important to study the risk factors of myopia for myopia prevention and control. The current research suggests that simple gene changes are not enough to explain the rapidly increasing incidence of myopia In addition, researchers have provided evidence that environmental factors are also risk factors for myopia [6]. Thus, myopia is a complex disease caused by a combination of genes and environment [[7], [8], [9]]. This article briefly reviews the effects of the interaction between gene and environmental factors on the development of myopia.

## 2 Heredity and myopia

### 2.1 Genes related to myopia

Genetic factors play an essential role in the occurrence and development of high myopia. An increasing number of studies reveal the importance of genetic contributions [10]. The genetic factors controlling circadian rhythm and pigmentation are associated with the development of myopia and ametropia [11]. It is confirmed that MTOR and PDGFRA are related to the severity of myopia, possessing gene-gene interaction [12]. Various GJD2 genotypes have different effects on the development of myopia [13,14]. Researchers suggest that PDE4B may be a novel high myopia susceptibility gene and play an important role in the development of myopia [15,16]. Flitcroft DI and colleagues [17] found 21 new candidate myopic genes, including ADAMTS18, ADAMTS2, ADAMTSL4, AGK, ALDH18A1, ASXL1, COL4A1, COL9A2, ERBB3, FBN1, GJA1, GNPTG, IFIH1, KIF11, LTBP2, OCA2, POLR3B, POMT1, PTPN11, TFAP2A, and ZNF469. In addition, FLRT3/SLC35E2B [18] and MYP2 [19] may represent candidate genes of myopia, which are related to the pathogenesis of high myopia (H.M.). Simpson CL and colleagues [20] found that WISP1 and SSPO are potential pathogenic genes in Ashkenazi Jewish families. Meguro A and colleagues [21] found HIVEP3, NFASC/CNTN2, CNTN4/CNTN6, FRMD4B, LINC02418, AKAP13 are associated with HM. In European individuals, FAM150B-ACP1, LINC00340, FBN1, DIS3L-MAP2K1, ARID2-SNAT1, and SLC14A2 are associated with refractive error [22]. PIK3CG and PRKAR2B are significantly correlated with the susceptibility to myopia in chicks [23], suggesting the role of this locus in the susceptibility to myopia in the population.

Studies have shown that heterozygous LoF mutations in CPSF1 [24], ARR3 and NDUFAF7 mutations [25] are related to early onset H.M. Seven heterozygous mutations-p.Pro287Leu, p.Arg319Thr, and p.Arg84Trp from SLC39A5 p.Lys661Arg, p.Ala528Thr, p.Phe44Leu from LEPREL1, and p.Arg321 from LRPAP1 were found in the study of Chinese cohort of high myopia patients, and p.Pro287Leu, p.Arg319Thr, p.Lys661Arg were identified as potential pathogenic mutations among them [26]. TNFRSF21 mutation can lead to nonsyndromic H.M. in Chinese people [27]. Zheng, Y. H. and colleagues [28] found a mutation in AGRN, providing further evidence for the potential role of AGRN in H.M. inheritance. Moreover, P4HA2 mutation may lead to axial elongation of the myopia eyeball [29]. These findings expand the current genetic spectrum of H.M.

Recently, Simpson, C. L. and colleagues identified a significant chain peak of myopia in African American families at 7p15.2 and 7p14.2 [30]; meanwhile, Musolf AM et al. [31] also reported a significant genetic linkage peak of myopia in Chinese Han patients at 10q26. 13. In addition, researchers found 1p36.12, 8q24, 7q36.1, and 11p15.1 were related to myopia in Ashkenazi Jewish families [20]. These findings indicate that the genetic linkage peak is associated with the occurrence of myopia.

#### 2.1.1 Single nucleotide polymorphism and myopia

Researchers indicate that PAX6 rs644242 [32], ZC3H11B rs4373767 [33,34] and BICC1 rs7084402 [33,35], VIPR2 rs885863 [36] and rs6979985 [37], ZMAT4 rs7829127 [36], TNKS rs4840437 and rs6989782 [38], PDGFRA rs2114039 [39], SOX2 rs4575941 [40], FGF10 rs339501, rs2973644, and rs 79002828 [41] are associated with severe myopia (moderate and extreme myopia). RSPO1 SNP rs12144790 and WNT7B rs10453441 [42] are related to children's visual axis length. ZFHX1B rs13382811 [34,43], PTPN5 rs1550870 and RASGRF1 rs6495367 [44], TGF receptor one rs10760673, and TGF receptor 2-AS1 rs7550232 [45] are also bound up with myopia in children. MMP-9 rs2236416 is interrelated to myopia in the population, suggesting that the MMP-9 gene locus may play a role in myopia [46]. Moreover, 4q25 rs10034228, 15q14 variation rs524952, and MIPEP variation rs9318086 were significantly concerned in myopia and different myopia severity in southern Chinese people, which together increased the susceptibility risk of high myopia by ten times [47] (Table 1).

**Table 1:** Genes related to myopia.

Genes/Gene mutation/Chain peak/Single Nucleotide	First Author	Publication Date	Reference
MTOR, PDGFRA	Li, X.	2022	[12]
GJD2	Solouki, A.M.	2010	[14]
PDE4B	Zhao, F.	2021	[15]
	Zhao, F.	2022	[16]
ADAMTS18, ADAMTS2, ADAMTSL4, AGK, ALDH18A1, ASXL1, COL4A1, COL9A2, ERBB3, FBN1, GJA1,GNPTG, IFIH1, KIF11, LTBP2, OCA2, POLR3B, POMT1, PTPN11, TFAP2A, ZNF469	Flitcroft, D. I.	2018	[17]
FLRT3/SLC35E2B	Swierkowsk, J.	2021	[18]
MYP2	Rasool, S.	2021	[19]
WISP1, SSPO	Simpson, C.L.	2019	[20]
HIVEP3, NFASC/CNTN2, CNTN4/CNTN6, FRMD4B, LINC02418, AKAP13	Meguro, A.	2020	[21]
FAM150B-ACP1, LINC00340, FBN1, DIS3L-MAP2K1, ARID2-SNAT1, SLC14A2	Fan, Q.	2016	[22]
PIK3CGPRK, AR2B	Huang, Y.	2019	[23]
CPSF1	Ouyang, J.	2019	[24]
ARR3, NDUFAF7 mutations	Liu, F.	2020	[25]
p.Pro287Leu, p.Arg319Thr and p.Arg84Trp from SLC39A5; p.Lys661Arg, p.Ala528Thr, p.Phe44Leu from LEPREL1, and p.Arg321His from LRPAP1	Feng, C. Y.	2017	[26]
TNFRSF21	Pan, H.	2019	[27]
AGRN	Zheng, Y. H.	2021	[28]
P4HA2	Napolitano, F.	2018	[29]
7p15.2 and 7p14.2	Simpson, C.L.	2021	[30]
10q26. 13.	Musolf, A. M.	2018	[31]
1p36.12, 8q24, 7q36.1 and 11p15.1	Simpson, C.L.	2019	[20]
PAX6rs644242	Tang, S. M.	2018	[32]
ZC3H11B rs4373767	Li, F. F.	2021	[33]
	Tang, S. M.	2020	[34]
BICC1 rs7084402	Li, F.	2017	[35]
VIPR2 rs885863, ZMAT4 rs7829127	Cheong, K.X.	2020	[36]
VIPR2 rs6979985	Zhao, F.	2022	[37]
TNKS rs4840437 and rs6989782	Jiang, L.	2019	[38]
PDGFRA rs2114039	Jiang, L.	2022	[39]
SOX2 rs4575941	Li, L.	2021	[40]
FGF10 rs339501, rs2973644, rs 79002828	Sun, W.	2019	[41]
RSPO1 rs12144790, WNT7B rs10453441	Lu, S. Y.	2020	[42]
ZFHX1B rs13382811	Li, J.	2017	[43]
PTPN5 rs1550870, RASGRF1 rs6495367	Xiao, H.	2021	[44]
TGF receptor one rs10760673, TGF receptor 2-AS1 rs7550232	Liu, L.	2021	[45]
MMP-9 rs2236416	Li, Y.	2022	[46]
4q25 rs10034228, 15q14 rs524952, MIPEP rs9318086	Liu, J.	2021	[47]

#### 2.1.2 DNA methylation and myopia

A large number of studies have shown that DNA methylation plays a vital role in the occurrence and development of myopia [48,49]. IGF-1 gene methylation may play a role in the pathogenesis of form deprivation myopia (FDM) in guinea pigs [50]. LINE-1 hypermethylation may be related to high myopia in humans and mice [51]. Furthermore, studies have shown that reducing DNA methylation levels is a risk factor for early-onset myopia in children [52]. The above results provide us with new strategies to intervene in myopia.

### 2.2 Non-coding RNA and myopia

Non-coding RNAs include microRNAs (miRNAs), repetitive RNAs, intronic RNAs, and long non-coding RNAs (lncRNAs), which are a diverse group of biomolecules with broad potential to control gene expression [53]. It is shown that mmu-mir-1936, mmu-mir-338-5p, mmu-mir-673-3p [54], miR-328 [55], miR-708a, and miR-148 [56] are related to myopia and can be used as biomarkers of myopia. MicroRNA-29a may affect myopia development by regulating type I collagen [57]. In addition, hsa-miR-142-3p in the aqueous humor is positively correlated with visual axis length [58]. The intervention of choroidal vascular dysfunction by adjusting circRNA-FoxO1 level is a potential strategy to prevent and treat myopia [59]. Studies have found that collagen metabolism affects the mechanical properties and remodelling of the sclera, thereby affecting the sclera's growth, axial elongation, and even myopia [60]. In the myopic group, the sclera expression of miR-29a, miR-29b, miR-29c, and MMP2 were significantly increased, while the sclera expression of COL1A1 was significantly decreased. Sclera soaked in 1 % genipin at 24 °C and a humidity of 40 % for 4h reverses these effects in myopia to achieve the purpose of treating myopia [61]. Guo D et al. [62] identified 27 differentially expressed MicroRNAs in the sclera of guinea pigs with myopia induced by the negative lens, 10 of which were up-regulated and 17 of which were down-regulated, and miR-19b-3p participated in the development of myopia by regulating the metabolic process. So far, a large number of studies have enriched the expression profiles of miRNAs [[63], [64], [65], [66]] and lncRNA [67], which may open a new field of vision for the pathogenesis and biology of myopia.

## 3 Environment and myopia

### 3.1 Proximal factors

#### 3.1.1 Near work

In terms of human beings, near work significantly reduces choroidal blood perfusion (ChBP), which might cause scleral hypoxia, leading to myopia [68]. The decrease of ChBP leads to scleral hypoxia and the transdifferentiation of scleral myofibroblast, α-Smooth muscle actin increased expression, eventually leading to the development of myopia [69]. Increased ChBP alleviates scleral hypoxia, inhibiting myopia development [70]. In addition, our research showed that electroacupuncture can improve choroidal vascular density, which may improve scleral hypoxia and inhibit sclera growth in myopia guinea pigs [71].

Near work refers to working distances shorter than 30 cm [72,73]. Currently, a lot of reading and digital screen activities (e.g., computers, video games, and the internet) can be seen as forms of near work. Sun JT and colleagues [74] indicated that working closely for more than 30 min without resting for 5 min is more likely to cause myopia. Saxena R et al. [75] showed that reading/writing for more than 6 h per day and video games for more than 4 h per week are risk factors for myopia. Therefore, reading or playing video games for long time may cause myopia. Nevertheless, video games are more likely to cause myopia than reading, possibly because the small size and high resolution of smart devices lead to high pixel density, requiring a very close viewing distance to enlarge the retinal image and parse fine details [76] and suggesting that preventing the progression of myopia may be achieved by enlarging the size of smart devices to increase the working distance.

#### 3.1.2 Outdoor activities

A large number of studies have shown that less outdoor activity is a risk factor for myopia [77], while increased outdoor activities have a protective effect on myopia [74,75,78,79]. Outdoor activities lasting more than 2 h per day have a protective effect on myopia [38,75]. Enthoven CA and colleagues [80] indicated that outdoor exposure exceeding 7 h per week is necessary to compensate for low-intensity near work, while outdoor exposure exceeding 14 h per week is necessary to compensate for preventing moderate or high-intensity near work. However, this protective effect is limited to the onset of myopia, not the progression of myopia after it has been diagnosed [81]. Interestingly, a study on myopia in the offspring of rural children in Handan City found that outdoor activities have a weaker protective effect on myopia in children [82]. In addition, studies have shown that puberty may play a regulatory role in the relationship between outdoor time and refractive development of Chinese children and adolescents [83], which provides clues for in-depth mechanism explanation and effective intervention strategies. Reducing the risk of wearing glasses is related to more exposure to green space [84], providing a new idea for us to intervene in myopia. These findings show that outdoor activities are of great significance for controlling myopia, indicating that people may prevent myopia by increasing the time spent on outdoor activities.

#### 3.1.3 Light

Dopamine is an essential neurotransmitter in the retina, which mediates various functions, including retinal development, visual signal transmission, and refractive development [85]. Bright light (2500–5000lx) inhibits the development of FDM by activating the dopamine D1 receptor signalling in the ON pathway in the retina [86]. The dopamine D1 receptor plays a key role in apomorphine-induced inhibition of FDM in mice [87]. Researchers showed that 120 min/day of outdoor lighting in schools is beneficial to restrain myopia [88], while lack of sunlight may cause myopia to be deepened [89]. Exposure to brighter light (400–40000lx) can reduce the risk of myopia [90]. Low ambient lighting level (∼50lx) reduces the effectiveness of the visually dependent mechanism regulating refractive development [91]. Using LED lights for work and dim lights are possible risk factors for myopia [92]. Hu YZ et al. [93] found that compared with high colour temperature light (4000 and 5000 K), low correlated colour temperature (CCT) light slowed down the axial growth of young monkeys. This study provides a new method for preventing and treating children's axial hyperelongation myopia. Thus, creating a low colour temperature (2700 and 3000 K) lighting environment in classrooms and other learning places may effectively protect children's vision [90].

In addition, researchers have also found that purple light [[94], [95], [96]] can inhibit myopia and is a preventive strategy to prevent the development of myopia. Colour cues play a role in visual-dependent emmetropia in primates. Narrowband and long wavelength illumination may prevent axial elongation, usually caused by form deprivation or hyperopia defocusing, by generating directional signals that are usually associated with nearsightedness defocusing [97].

#### 3.1.4 Classroom environment

Zhang, X. Y. et al. [98] found that compliance with school environmental monitoring indicators has a protective effect on the occurrence and development of myopia among students. For example, the per capita area is 1.36∼m2, the uniformity of the desk is 0.40–0.59, the average reflection ratio of the blackboard is 0.15–0.19, the uniformity of the blackboard is 0.80∼, and the average illumination of the blackboard is 150∼, 300∼, and 500∼lx, which are protective factors for the length of the eye axis. The uniformity of the blackboard between 0.40 and 0.59 is a risk factor for eye axis length. The average illuminance of 150∼, 300∼, and 500∼lx is a protective factor for the diopter, while the average illuminance of 500∼lx on the desk is a protective factor for the diopter. The pilot study on new classrooms by Zhou, Z. et al. [99] has made it possible to prevent and control the occurrence and development of myopia by widely using bright classrooms instead of traditional ones.

### 3.2 Distal factors

#### 3.2.1 Education and economy

The prevalence of myopia may be related to the level of economic development. Researchers found that the prevalence rate of children in rural and suburban areas was significantly lower than that of students of the same age living in urban areas with highly recognized socioeconomic status [100]. Economic development may increase the desire to pursue wealth. Many individuals may pursue wealth through education, resulting in heavier education burdens and longer education years, increased working hours, and reduced outdoor time, eventually leading to myopia's prevalence. In turn, higher levels of education may promote economic development, which may aggravate this process [101]. In addition, the increase in educational pressure may promote the development of myopia. Researchers found that the longer the education years, the higher myopia prevalence [102]. In the United States, myopia is related to higher education [103]. These findings show that the development of myopia is related to the level of education and economic development. Among them, the impact of economic development level on myopia may be caused by a combination of factors.

#### 3.2.2 Population density

Zhang XY et al. [98] found the per capita area of 1.36∼m^2^ is a protective factor for the length of the eye axis. The study found that children's axial length and childhood refractive error were related to high population density and small family size. A narrow living space may be an environmental threat to children's myopia development [104]. The defocusing profile in the family environment, especially in the near central field of vision, is related to the result of refractive errors in childhood and is likely to be a potential risk factor for myopia [105]. It has shown that urban and indoor environments are similar to the spatial frequency components known to induce form deprivation myopia in animal models, which are potential risk factors for myopia development [106].

To sum up, near work, outdoor activities, education, light, economy, education, classroom environment, population density, and so on are environmental factors that affect myopia.

## 4 Gene-environment interaction and myopia

It is generally believed that myopia results from the interaction of genes and environment [4], and myopia's gene-environment (GxE) or gene-gene interaction is widespread [107]. The GxE interaction of myopia can be defined as “different effects of environmental exposure on the myopia of different candidate genetic factors” or equivalent to “different effects of candidate genes on myopia of different environmental exposure [108]. By calculating the values of the environment and genetic index to prove the influence of genetic and environmental interaction on myopia, it is found that the calculated value of the environmental and genetic index (EGI) is 0. 125, indicating that genetic factors may play a role of 12.5 % in the formation of myopia, and environmental factors may play a role of 87.5 % in the formation of myopia [109]. This research result shows that myopia is not caused by environmental factors or genetic factors alone but by the joint action of environment and genes. Enthoven CA and colleagues [110] showed that environment and heredity are related to myopia, and their combined effects have the greatest impact; that is, GxE interaction is significantly related to myopia. Researchers found that genetically susceptible individuals may develop myopia if exposed to certain environmental factors [111]. Reducing high-risk environmental factors that affect myopia may benefit genetically susceptible individuals with myopia due to environmental factors, effectively decreasing myopia's prevalence and providing us with a new plan to prevent and control myopia. There is a stable interaction between rs7829127 in ZMAT4 and near-work activities [112], providing evidence support for GxE interaction. In addition, the study also found that increasing near work duration and reducing outdoor exposure in childhood significantly enhanced the role of myopia genes [10]. Multivariate logistic regression analysis showed that, for children and adolescents aged 6–14 years in the low-risk population of genetic risk score (GRS), compared with those who worked continuously for less than half an hour, those who worked continuously for more than half an hour had a higher risk of myopia; In the moderate risk group of GRS, the risk of myopia increases with the increase of daily use of computers; In the high-risk population of GRS, the risk of myopia increases with the increase of total daily reading and writing time [113], which indicates that gene-environment interaction has an important impact on myopia. Using smart devices is more likely to cause myopia than paper printed materials, not because of the device itself, but because long-term viewing of handheld electronic devices with high-resolution displays requires a very close viewing distance [76]. However, prolonged reading may also exacerbate the myopia progression [114]. Therefore, whether using electronic products or paper prints, it is necessary to adopt th e right way, such as increasing the working distance and outdoor exposure. The differential expression of ametropia is related to the reading time of those with a low-frequency variation of APLP2, a myopic susceptibility gene [115], which provides evidence support for the hypothesis of GxE interaction in the development of myopia for a long time. The above research results show that the interaction between environmental factors and genes represented by near work has an important impact on myopia, which provides evidence to support intervention in myopia through near work. The interaction between genes and education in the adult cohort shows that higher education levels have more significant genetic effects than lower education levels [116,117]. Researchers found that the rs2969180 Aallele of SHISA6-DNAH9, the rs524952 A allele of GJD2, and the rs2137277 A allele of ZMAT4-SFRP1 are strongly associated with myopia in individuals who have received higher education [118]. The three genome-wide significant loci AREG, GABRR1, and PDE10A show a strong interaction with education in the Asian population, and individuals with higher education levels have greater genetic effects than those with lower education levels.

In contrast, the interaction is not obvious in Europeans [22]. The different effects of education intensity on childhood onset and high myopia are amplified over time, indicating that the cohort effect in gene-environment interaction may increase the prevalence of myopia if the increase of childhood education intensity is not inhibited [119]. Recently, Clark R and colleagues [120] found that education interacts with genetic variants near GJD2, RBFOX1, LAMA2, KCNQ5, and LRRC4C to contribute to myopia susceptibility. These results indicate that the interaction between environmental factors represented by education and genes has an essential impact on myopia, providing a new idea for controlling the development of myopia (Table 2).

**Table 2:** Interaction between heredity and environmental factors in the development of myopia.

Genetic Factors	Environment Factors	First Author	Reference
Heredity	Near work (homework time and staring at an electronic screen)	Zhang, X.	[109]
High genetic risk score (high GRS)	Near work and Outdoor activities	Enthoven, C.A.	[110]
rs7829127 in ZMAT4	Near work	Fan, Q.	[112]
GRS	Time of near work	Liu, S. X.	[113]
APLP2 rs188663068	Time spent reading	Tkatchenko, A.V.	[115]
Genetic predisposition	Education	Verhoeven, V.J.	[117]
SHISA6-DNAH9: rs2969180 A allele, GJD2: rs524952 A allele, ZMAT4-SFRP1: rs2137277 A allele	Education	Fan, Q.	[118]
GJD2, RBFOX1, LAMA2, KCNQ5, LRRC4C	Education	Clark, R	[120]

## 5 Intervention approaches for myopia

At present, studies have shown that orthokeratology [121], soft contact lenses [122], repeated low-level red light therapy [123], and low concentration atropine [124] can effectively slow myopia progression and axial elongation. The study also showed that orthokeratology combined with 0.01 % atropine was more effective than sole orthokeratology in delaying myopia development [125]. In addition, 7-MX, a non-selective adenosine antagonist, may also have therapeutic potential for slowing the progression of myopia in humans [126].

## 6 Conclusion

In conclusion, near work, outdoor activities, light, classroom environment, education and economy, and population density are risk environmental factors for myopia. Both gene and environmental factors play an essential role in the occurrence and development of myopia.Thus, interaction between gene and environmental factors has the greatest impact on the development of myopia. To prevent myopia progression, reducing near work, increasing outdoor activities, improving the classroom environment and so on may be appropriate methods for controlling myopia, in addition, improving the interaction between genes and environmental factors by regulating genes and environmental factors is of great importance in controlling myopic development.

## Ethics statement

Review and approval by an ethics committee was not needed for this study because this was a literature review, and no new data were collected and analyzed. For the same reason, informed consent was not required.

## Funding

This study was supported by the National Key R&D Program of China (2021YFC2702103, 2021 YFC2702100) and the Key R&D Program of Shandong Province (2019GSF108252).

## Data availability statement

All data to support the conclusions have been publicly available in the main test of all references.

## CRediT authorship contribution statement

**Jiawen Hao:** Writing – original draft, Conceptualization. **Zhaohui Yang:** Investigation. **Ruixue Zhang:** Investigation. **Zhongyu Ma:** Visualization. **Jinpeng Liu:** Visualization. **Hongsheng Bi:** Writing – review & editing. **Dadong Guo:** Writing – review & editing, Conceptualization.

## Declaration of competing interest

The authors declare that they have no known competing financial interests or personal relationships that could have appeared to influence the work reported in this paper.
